# Supplementary material for: Measuring the olfactory bulb input-output transformation reveals a contribution to the perception of odorant concentration invariance
Source: Nat Commun. 2017 Jul 19;8:81. doi: 10.1038/s41467-017-00036-2 (PMC5517565; doi:10.1038/s41467-017-00036-2)
Supplement: Supplementary file 1 — Supplementary Information [file 41467_2017_36_MOESM1_ESM.pdf]

File Name: Supplementary Information

Description: Supplementary Figures and Tables

File Name: Peer Review File
Description:

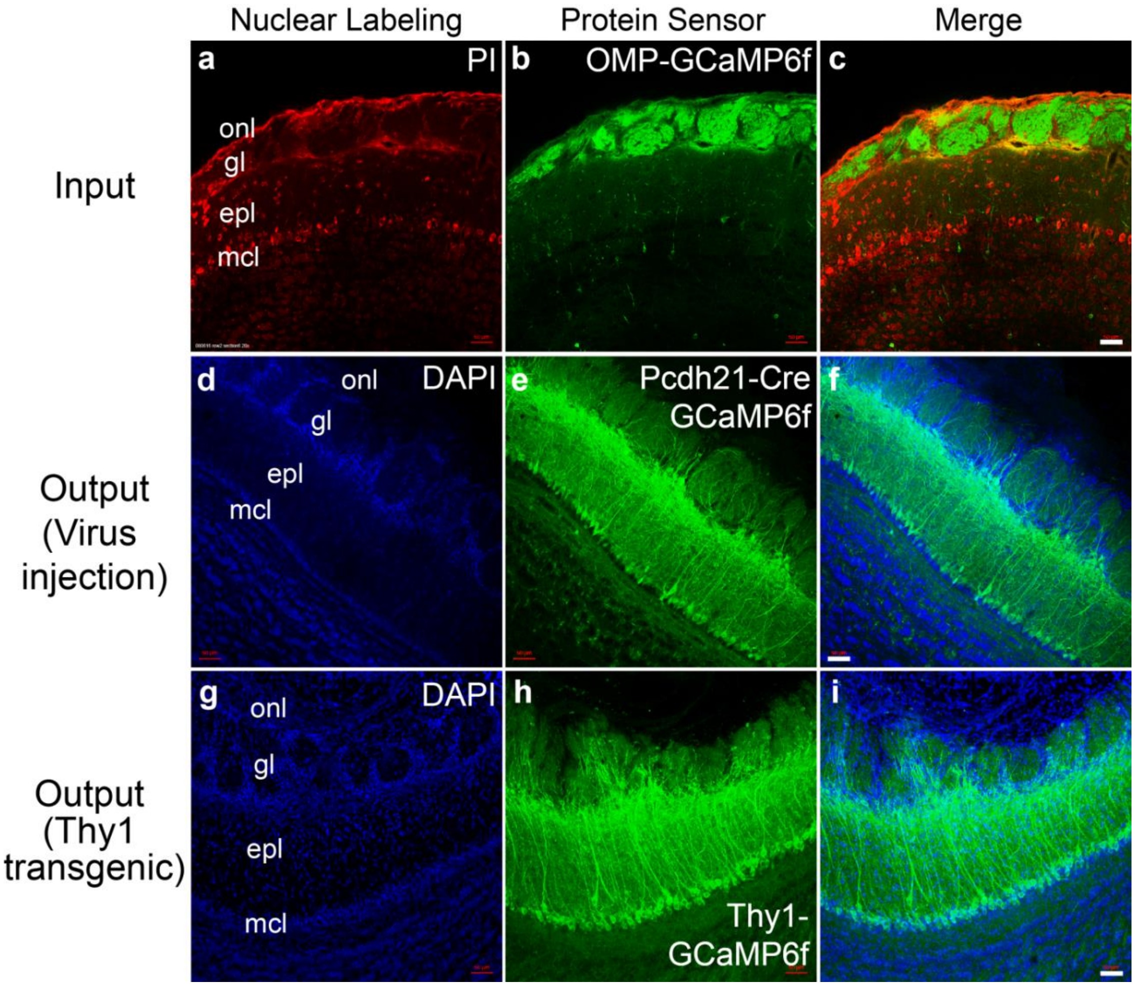

Supplementary Figure 1: Histological examination of expression patterns of GCaMP6f in the input and output. (**a**-**c**) GCaMP6f expression in an OMP-GCaMP6f transgenic mouse. (**d**-**f**) Virally expressed GCaMP6f in a Pcdh21-Cre transgenic mouse. (**g**-**i**) Transgenic mouse (Thy1-GCaMP6f) expression of GCaMP6f in mitral and tufted cells. PI, Propidium Iodide. OMP, olfactory marker protein; onl, olfactory nerve layer; gl, glomerular layer; epl, external plexiform layer; mcl, mitral cell layer. Scalebars in **c**, **f**, and **i** are 50 µm.

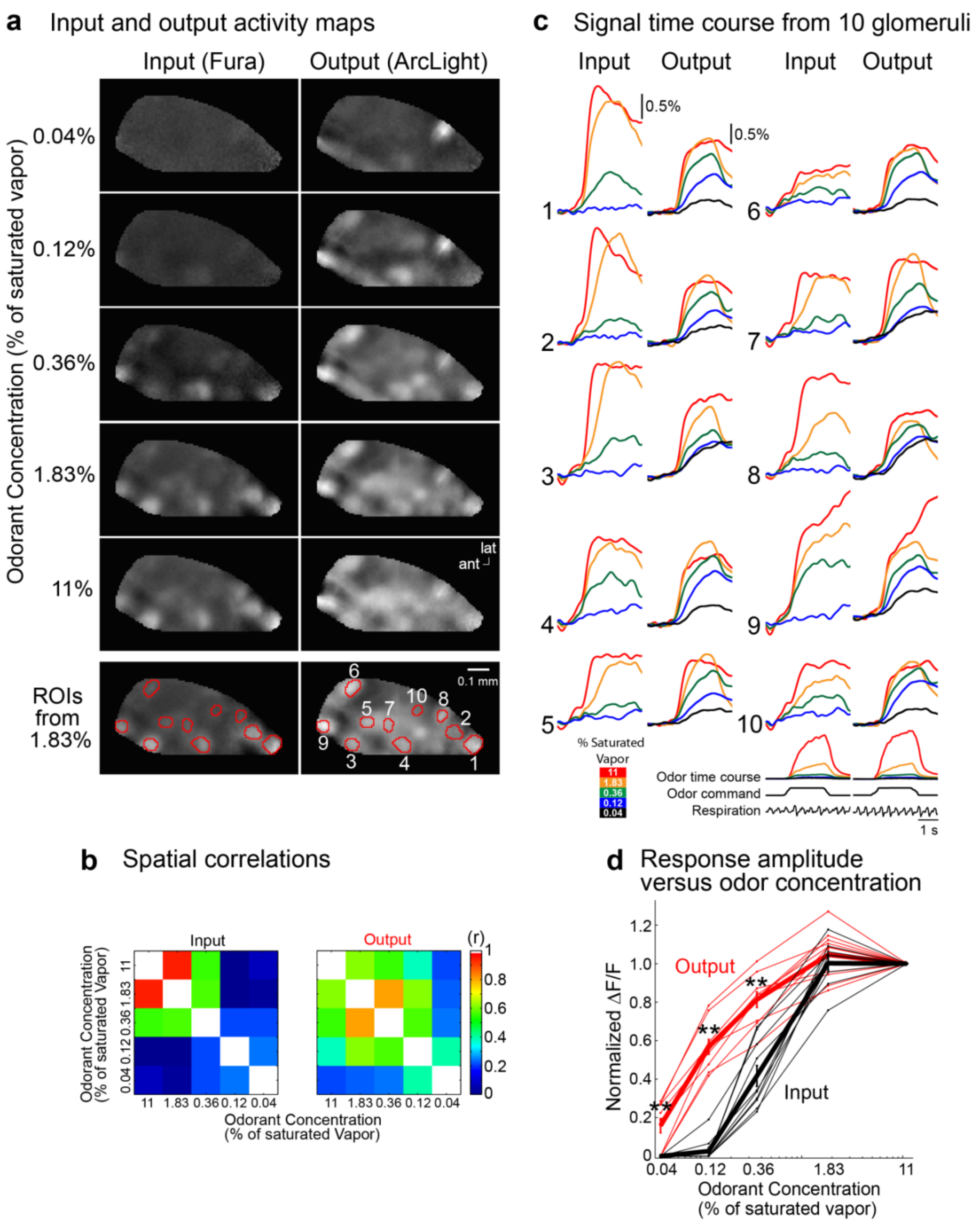

Supplementary Fig. 2: Comparing the input and output from 10 glomeruli in the same bulb using Fura dextran (input) and the voltage sensor ArcLight (output). (**a**) Frame subtraction maps of activity at five odorant (methyl valerate) concentrations for the input (left) and output (right). The output maps are much more similar to each

other in comparison to the input maps. The bottom panel (ROIs from 1.83%) indicates the selected glomeruli (red overlay) for the time course results in panel **c**. (**b**) Spatial correlation of the frame subtraction map from each odorant concentration with every other concentration. (**c**) Input and output traces of fluorescence versus time for 10 glomeruli. The input signals decrease more dramatically than those of the output. Input and output measurements were performed alternatively. Fura dextran was imaged using 380 nm excitation light and emission was measured using a 510/84 nm band-pass filter. ArcLight was imaged using 480 nm excitation light and a 535/40 nm band-pass emission filter. The traces are low-pass filtered at 1 Hz. Odor time course (measured simultaneously with a photo-ionization detector), odor command pulse, and respiration are shown under glomerulus 10. The odor time course was taken from the aligned averages. The respiration trace is from a single trial. (**d**) Normalized peak fluorescence change versus odorant concentration for the glomeruli in **c** (black: input; red: output). The activity maps in panel **a** and the traces in panel **c** are the average of 4-20 individual trials aligned to the first sniff following odor onset. The activity maps in **a** are spatially high-pass filtered for display purposes, but the correlation analysis in **b** was carried out on unfiltered maps that were corrected for an exponential drift. The error bars represent s.e.m., $^{**}p < 0.001$ (Wilcoxon rank sum test). ant, anterior; lat, lateral. The 0.04% odor concentration did not evoke any input glomerular response, and was subtracted from the input for the traces and values in **c** and **d**. Detailed statistical results are in **Supplementary Table 2**.

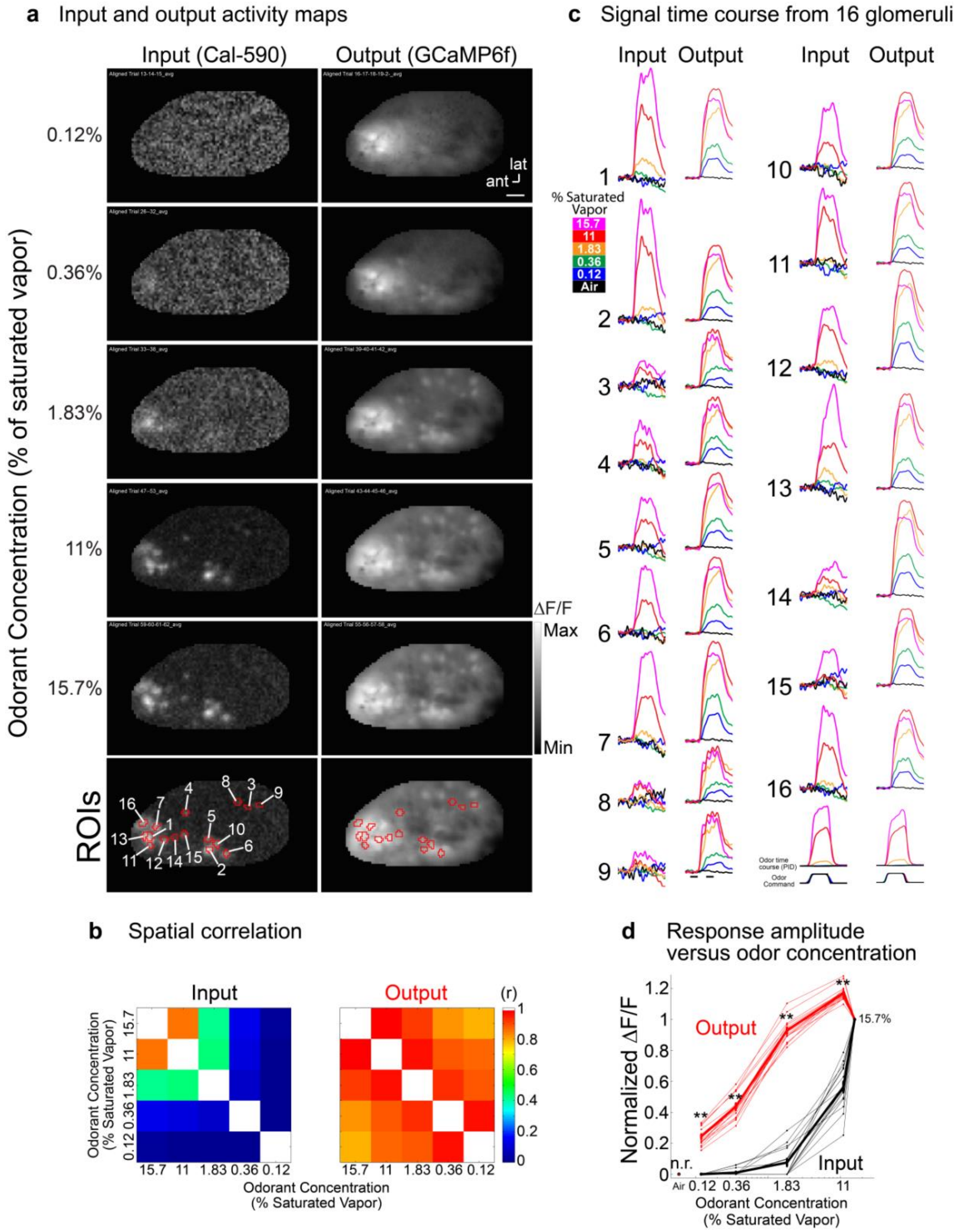

Supplementary Fig. 3. Comparing the input and output from 16 glomeruli in the same bulb using Cal-590 dextran (input) and GCaMP6f (output) in a Thy1-GCaMP6f transgenic mouse. (**a**) Frame subtraction maps of activity at 5 odorant (methyl valerate) concentrations for the input (left) and output (right). The output maps

are much more similar to each other than are the input maps. The bottom panel (ROIs) indicates the selected glomeruli (red overlay) for the time course results in panel **c**. The largest glomerular signals (ΔF/F) for each map are from low to high concentration: (*input*) 0%, 0.2%, 0.5%, 2.1%, 3.1%; (*output*) 12%, 20%, 36%, 43.7%, 37%. (**b**) Spatial correlation of the frame subtraction map from each odor concentration with the map evoked by the other concentrations. (**c**) Input and output traces of fluorescence versus time for 16 glomeruli. The odor time course was measured by placing a PID next to the mouse's nose during recordings. (**d**) Normalized peak fluorescence change versus odorant concentration for the glomeruli in **c** (black: input; red: output). The evoked signal size for each concentration was normalized to the signal evoked at 15.7% of saturated vapor. Input and output measurements were performed alternatively using Cal-590 dextran and GCaMP6f using 572/23 nm and 479/39 nm band-pass excitation filters. The activity maps and traces are from aligned averages of 4-7 individual trials. The black bars underneath the output trace for glomerulus 9 indicates the time points used to generate the activity maps in panel **a**. The error bars represent s.e.m., $^{**}p < 0.001$ (Wilcoxon rank sum test). Detailed statistical results are in **Supplementary Table 2**. For the population summary in **Fig. 3b** and **Supplementary Table 2**, the signal size was normalized to 11%. Scale bar in **a**, 250 µm. ant, anterior; lat, lateral.

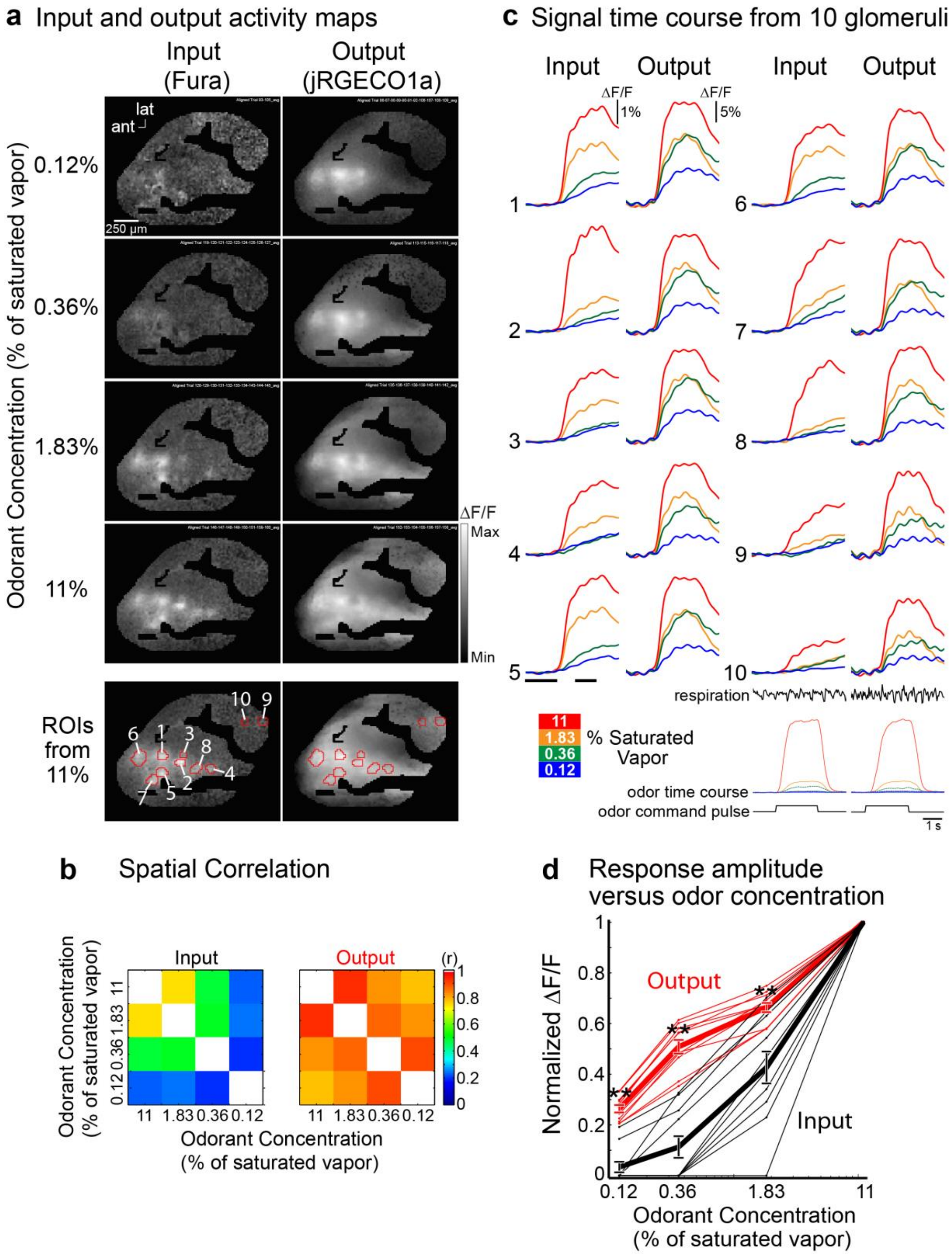

Supplementary Fig. 4. Comparing the input and output from 10 glomeruli in the same bulb using Fura dextran (input) and jRGECO1a (output). (**a**) Frame subtraction maps of activity at four odorant (methyl valerate) concentrations for the input (left) and output (right). The output maps are much more similar to each other than are

the input maps. The bottom panel (ROIs from 11%) indicates the selected glomeruli (red overlay) for the time course results in panel **c**. The largest glomerular signals (ΔF/F) for each map are from low to high concentration: (*input*) 0.8%, 1.2%, 2.4%, 4%; (*output*) 7.6%, 13.8%, 16.4%, 23%. (**b**) Spatial correlation of the frame subtraction map from each odorant concentration with every other concentration. (**c**) Input and output traces of fluorescence versus time for 10 glomeruli. The input signals decrease more dramatically than those of the output. Input and output measurements were performed alternatively using Fura dextran and jRGECO1a. Fura dextran was imaged using 380 nm excitation light and emission was measured using a 510/84 nm band-pass filter. jRGECO1a was imaged using 565 nm excitation light and a 610 nm long-pass emission filter. The traces are low-pass filtered at 1 Hz. Respiration, the odor command pulse, and the odor time courses (measured simultaneously with a photo-ionization detector) are included under glomerulus 10. The respiration and the command pulse traces are taken from single trials. The odor time course was taken from the aligned averages. The black bars underneath the input trace for glomerulus 5 indicate the time points used to generate the activity maps in panel **a**. (**d**) Normalized peak fluorescence change versus odorant concentration for the glomeruli in **c** (black: input; red: output). The evoked signal size for each concentration was normalized to the signal evoked at 11% of saturated vapor. The activity maps in panel **a** and the traces in panel **c** are the average of 5-13 individual trials aligned to the first sniff following odor onset. The error bars represent s.e.m., **$p < 0.001$ (Wilcoxon rank sum test). ant, anterior; lat, lateral. Detailed statistical results are in **Supplementary Table 2**.

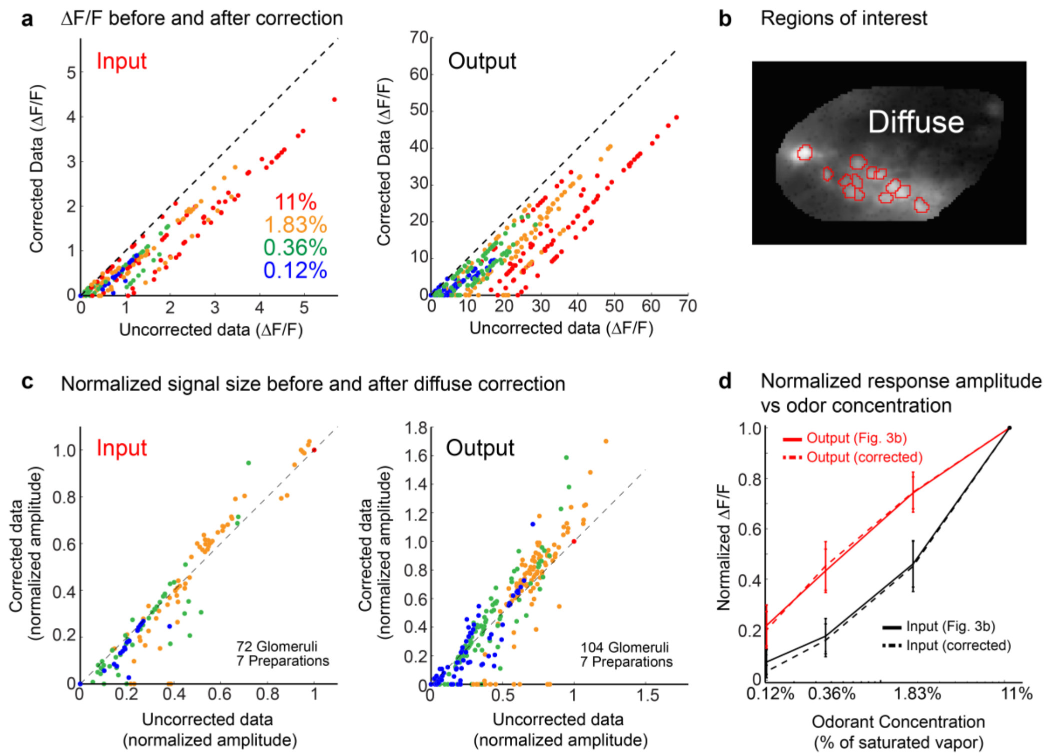

Supplementary Figure 5: **Correcting for a diffuse output signal does not significantly alter the concentration-response function of our input and output measurements**. (**a**) Individual input and output signal sizes values were corrected for a diffuse signal by subtracting the ΔF/F measured from regions of the dorsal bulb not exhibiting odor evoked glomerular peaks of activity from the ΔF/F measured from glomerular ROIs at each concentration. (**b**) Example of the glomerular ROIs and diffuse regions of interest used for the correction for the preparation in **Fig. 2**. (**c**) Corrected versus uncorrected signal sizes normalized to 11% of saturated vapor. (**d**) Input and output concentration response curves before (solid red) and after correction (dashed red). The original and corrected normalized signal sizes were not significantly different from each other at any concentration ($p > 0.62$ for all comparisons using a Wilcoxon rank sum test). The error bars represent s.e.m. The correction was performed in 7 preparations from **Fig. 3b** (72 input glomeruli and 104 output glomeruli).

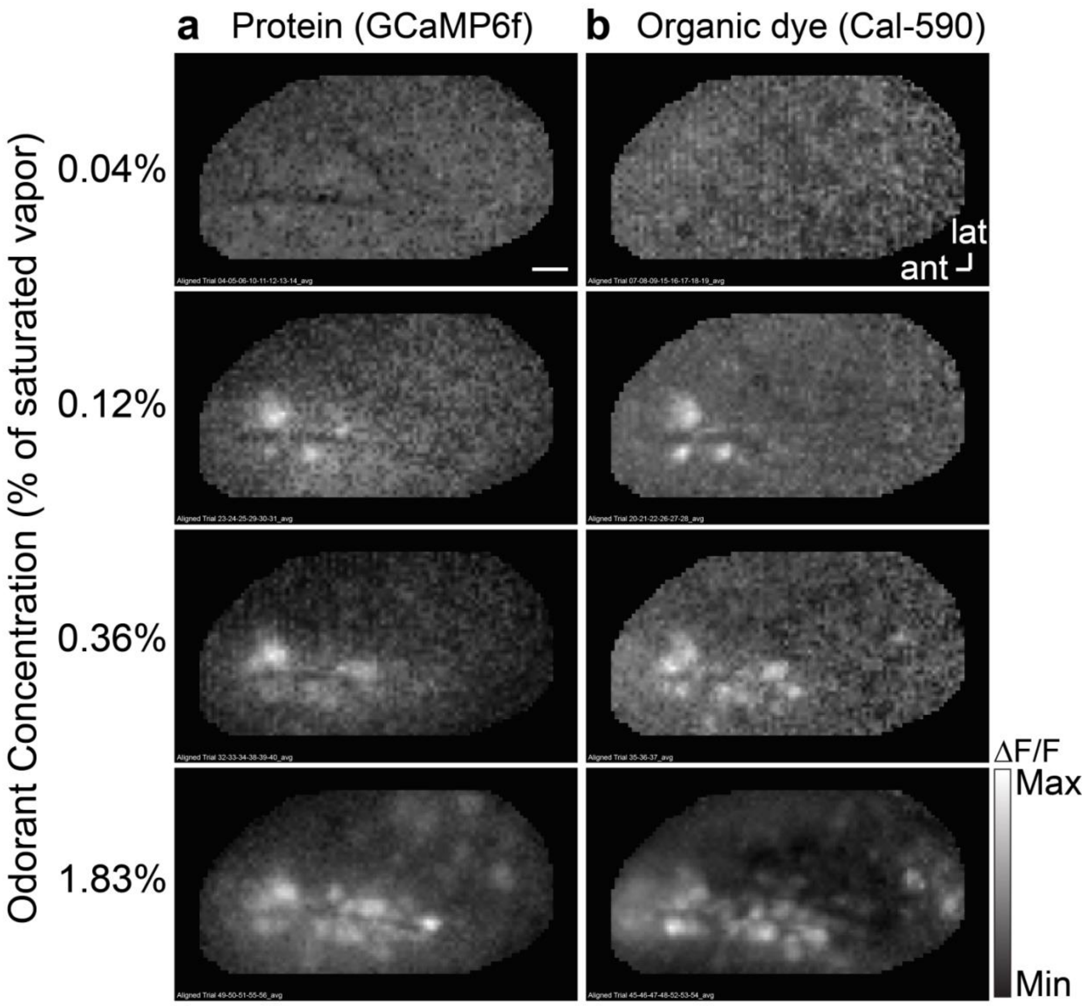

Supplementary Figure 6: Bulb input measured with a protein sensor and an organic calcium dye. (**a**) Input maps measured using GCaMP6f in OMP-GCaMP6f transgenic mice. (**b**) Input maps measured using Cal-590 dextran from the same hemibulb as in panel **a**. The dye bleached rapidly during recordings which made it impossible to quantify the relationship between the two sensors. However, the input activation maps evoked by the two sensors are qualitatively similar. Scale bar in **a**, 250 µm. ant, anterior; lat, lateral.

a Map correlation data in Fig. 3a (14 Measurements)

Comparison	Input (Mean ± SEM)	Output (Mean ± SEM)	p-val (Wilcoxon rank sum)	Ranksum (Wilcoxon rank sum)	Z-Val (Wilcoxon rank sum)
11% and 1.83%	0.63±0.06	0.90±0.02	< 0.001	126	-3.5
11% and 0.36%	0.41±0.05	0.78±0.04	< 0.001	116	-4
11% and 0.12%	0.25±0.07	0.61±0.08	0.01	78	-2.6
1.83% and 0.36%	0.41±0.06	0.84±0.04	< 0.001	117	-3.9
1.83% and 0.12%	0.26±0.07	0.68±0.07	0.003	72	-2.9
0.36% and 0.12%	0.27±0.07	0.75±0.07	0.001	69	-3.1

b Correlation analysis restricted to glomerular ROIs

Comparison	Input (Mean ± SEM)	Output (Mean ± SEM)	p-val (Wilcoxon rank sum)	Ranksum (Wilcoxon rank sum)	Z-Val (Wilcoxon rank sum)
11% and 1.83%	0.6±0.08	0.88±0.03	0.002	116	-3
11% and 0.36%	0.34±0.05	0.75±0.06	< 0.001	108	-3.4
11% and 0.12%	0.21±0.05	0.58±0.08	0.01	78	-2.6
1.83% and 0.36%	0.43±0.07	0.87±0.03	< 0.001	97	-4
1.83% and 0.12%	0.28±0.08	0.68±0.07	0.003	72	-2.9
0.36% and 0.12%	0.32±0.09	0.78±0.08	0.003	72	-2.9

Supplementary Table 1: Correlation analysis details. (**a-b**) Statistical details for the correlation analysis performed on the entire map (**a,** data in **Fig. 3a**), and restricted to glomerular regions of interest (**b**). Statistical significance was assessed using a Wilcoxon rank sum test for each odor condition of each preparation. Spatially high-pass filtering the output maps did not significantly decrease the output correlations.

SB: Same bulb alternatively
OBS: Opposite bulb simultaneously
OBA: Opposite bulb alternatively
ET: Ethyl tiglate
MV: Methyl valerate
IA: Isoamyl acetate
n.t. not tested

					1.83%					0.36%					0.12%				
**Prep**	**Exp Type**	**Input Sensor**	**Output Sensor**	**Odor**	**Input**	**Output**	**P-Val**	**Rank sum**	**Zval**	**Input**	**Output**	**P-Val**	**Rank sum**	**Zval**	**Input**	**Output**	**P-Val**	**Rank sum**	**Zval**
**1**	SB	Fura (1)	ArcLight (1)	ET	0.57	0.8	NA	NA	NA	0.27	0.7	NA	NA	NA	0	0.55	NA	NA	NA
**2**	SB	Fura (10)	ArcLight (10)	MV	1±0.03	1.04±0.04	n.s.	95	-0.7	0.41±0.05	**0.81±0.04**	< 0.001	58	-3.5	0.03±0.01	**0.56±0.03**	< 0.001	55	-3.8
**3**	SB	Fura (13)	GCaMP6f (13)	MV	0.53±.02	**0.88±0.02**	< 0.001	91	-4.3	0.26±0.02	**0.65±0.04**	< 0.001	92	-4.3	0.12±0.02	**0.49±0.04**	< 0.001	91	-4.3
**4**	SB	Fura (10)	jRGECO1a (10)	MV	0.42±.06	**0.66±0.02**	0.006	68	-2.8	0.11±0.05	**0.51±0.03**	< 0.001	55	-3.8	0.03±0.02	**0.26±0.02**	< 0.001	55	-3.9
**4**	SB	Fura (3)	jRGECO1a (3)	IA	0.4±0.2	0.89±0.02	NA	NA	NA	0	0.69±0.04	NA	NA	NA	n.t.	n.t.	NA	NA	NA
**5**	SB	Cal-590 (16)	GCaMP6f (16)	MV	0.14±0.04	**0.79±0.01**	< 0.001	136	-4.8	0.02±0.01	**0.37±0.01**	< 0.001	136	-5	0	**0.2±0.01**	< 0.001	136	-5.1
**6**	OBS	OG488B (8)	GCaMP6f (14)	MV	0.33±0.02	**0.62±0.03**	< 0.001	39	-3.6	0.11±0.02	**0.28±0.02**	< 0.001	37	-3.7	0	**0.02±0.006**	0.02	64	-2.3
**7**	OBS	OG488B (9)	GCaMP6f (17)	MV	0.42±.04	**0.63±0.02**	< 0.001	52	-3.7	0	**0.19±0.03**	< 0.001	58.5	-3.5	0	0	NA	NA	NA
**8**	OBS	OG488B (8)	GCaMP6f (25)	MV	0.48±0.03	**0.61±0.03**	0.04	88	-2	0.16±0.01	0.23±0.02	0.2	105	-1.3	0.01	**0.04±0.007**	0.005	71	-2.8
**9**	OBA	CG1 (7)	GCaMP6f (17)	MV	0.34±0.03	**0.67±0.01**	< 0.001	28	-3.7	0.09±0.03	**0.31±0.02**	< 0.001	32	3.5	0	**0.18±0.01**	< 0.001	28	-3.8
**10**	OBA	CG1 (7)	GCaMP6s (13)	MV	0.51±0.04	0.81±0.1	0.18	56	-1.3	0.033±0.02	**0.97±0.07**	0.0003	28	-3.6	n.t.	0.43±0.04	NA	NA	NA
**11**	OBA	OG488B (11)	jRGECO1a (8)	MV	0.38±0.03	**0.74±0.03**	< 0.001	124	-3.6	0.23±0.02	**0.47±0.03**	< 0.001	122	3.4	n.t.	0.16±0.01	NA	NA	NA
**12**	OBA	OG488B (5)	jRGECO1a (11)	MV	0.41±0.05	**0.92±0.03**	0.002	15	-3.1	0.14±0.07	**0.85±0.05**	0.002	15	-3.1	0	**0.27±0.02**	0.002	15	-3.1
**13**	OBA	OG488B (16)	GCaMP6f (22)	MV	0.65±0.04	0.64±0.01	0.4	341	0.8	0.3±0.03	**0.50±0.02**	0.0002	184	-3.8	0.23±0.03	**0.29±0.01**	0.01	229	-2.4
**14**	OBA	OG488B (18)	GCaMP6f (34)	MV	0.62±0.02	0.66±0.02	0.08	385	-2.8	**0.37±0.02**	0.26±0.03	0.015	605	2.4	**0.21±0.02**	0.13±0.01	0.006	618	2.7
				Population Summary	0.48±0.05	**0.76±0.03**	< 0.001	139	-3.9	0.17±0.03	**0.52±0.06**	< 0.001	141	-3.8	0.05±0.02	**0.26±0.05**	0.002	103	-3

Supplementary Table 2: Fig. 3b signal size analysis details. Prep: Individual mouse preparations used in the population summary. Two different odorants were used in preparation 4, and the results were analyzed independently. Exp Type: Experimental type indicating whether the measurements were performed in the same bulb alternatively (SB), opposite bulbs simultaneously (OBS), or opposite bulbs alternatively (OBA). The number in the parenthesis in the input and output sensor columns indicates the number of glomeruli used in the analysis for that preparation. For same bulb preparations, glomeruli that were identified for both input and output at any odor concentration were included. For opposite bulbs simultaneously and opposite bulbs alternatively preparations, input or output glomeruli activated by 1.83% saturated vapor were included. Measurements were made from those glomeruli for each odorant condition, and the signal amplitudes are normalized to the signal size evoked by 11% of saturated vapor. The indicated values are the average of the normalized signal size values for all the glomeruli identified for that preparation. Statistical significance was assessed using a Wilcoxon rank sum test for each odor condition of each preparation. A value of zero indicates that no odor-evoked response was detected at that odor concentration. Preparation #14 is a counter example in which the input was significantly more concentration invariant than the output at 0.36% and 0.12% of saturated vapor. Significantly larger values of the input or output for each % of saturated vapor are bolded. Fura, Fura dextran, OG488B, Oregon Green 488 BAPTA-1 dextran; CG1, Calcium Green-1 dextran; ET, Ethyl Tiglate; MV, Methyl Valerate; IA, Isoamyl Acetate. The preparations used in **Figs. 1-2**, and **Supplementary Figs. 2-4** are 1, 3, 2, 4, 5, respectively.

	Input indicator	Type	Kd (nM)	Hill Coefficient	Reference
1	Fura dextran	Calcium	350	~1	52,53
2	Calcium Green-1 dextran	Calcium	190	~1	54,55,56
3	Oregon Green 488 BAPTA-1 dextran	Calcium	170	~1	54,56,57,58
4	Cal-590 dextran	Calcium	561	n/a	29
	**Output indicator**	**Type**	**Kd (nM)**	**Hill Coefficient**	**Reference**
1	ArcLight	Voltage	NA	NA	30
2	GCaMP6f	Calcium	375	2.7, 2.3	32,58
3	GCaMP6s	Calcium	144	2.9	32
4	jRGECO1a	Calcium	148	1.9	33,34

Supplementary Table 3: Sensor properties. The values were taken from the indicated references and the manufacturer's product sheet. NA, not applicable.
